# Supplementary figures and images for: The Effect of Climate Parameters on Sheep Preferences for Outdoors or Indoors at Low Ambient Temperatures
Source: Animals (Basel). 2020 Jun 13;10(6):1029. doi: 10.3390/ani10061029 (PMC7341328; doi:10.3390/ani10061029)

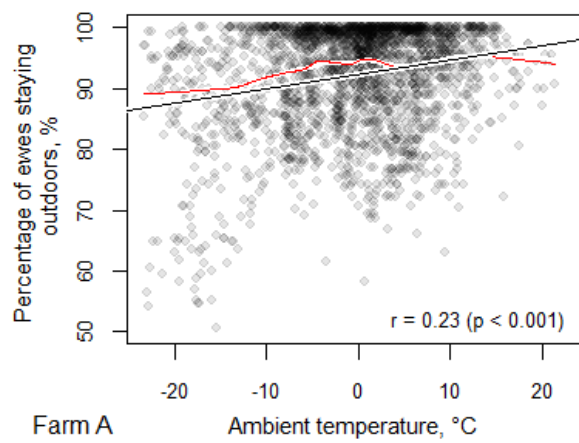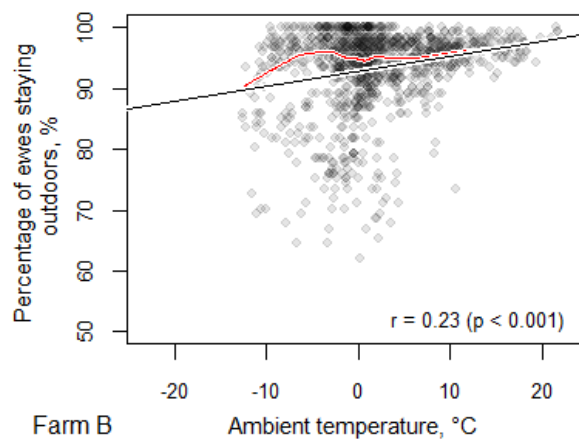

Supplement: Supplementary file 1 [file animals-10-01029-s001.zip › Supplementary/Figure S1.pdf]

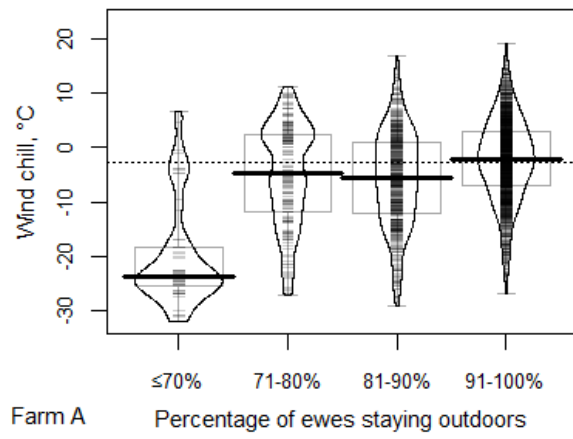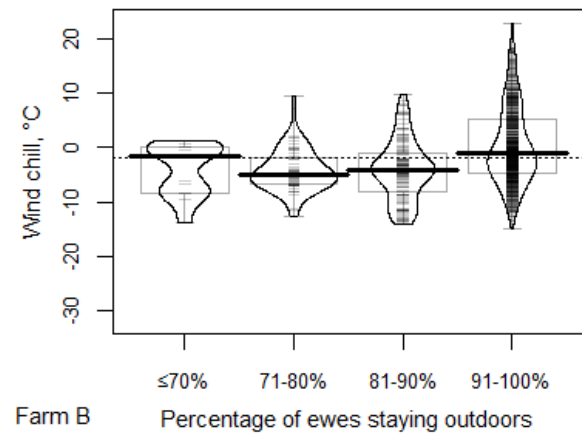

Supplement: Supplementary file 1 [file animals-10-01029-s001.zip › Supplementary/Figure S2.pdf]

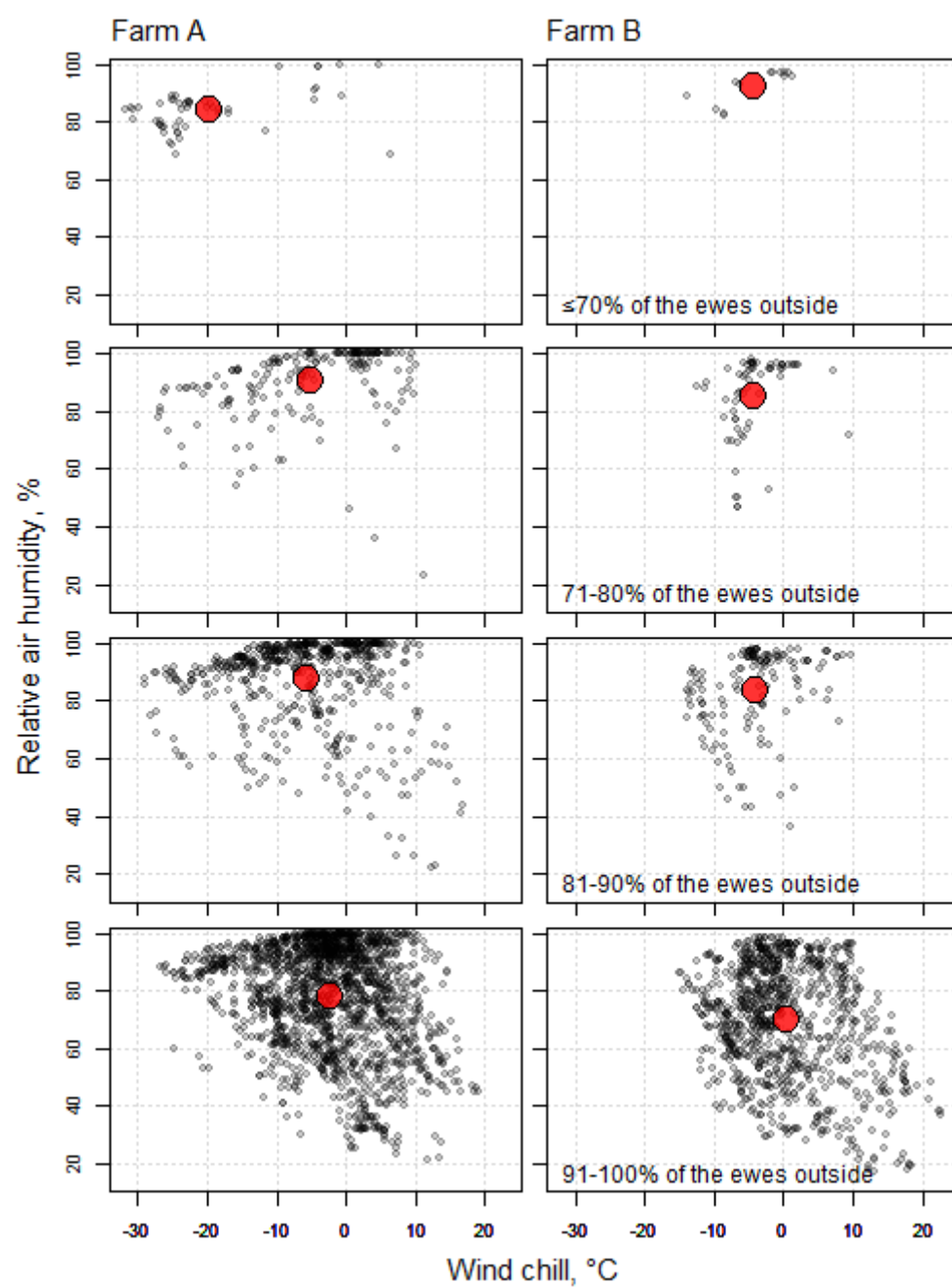

Supplement: Supplementary file 1 [file animals-10-01029-s001.zip › Supplementary/Figure S3.pdf]

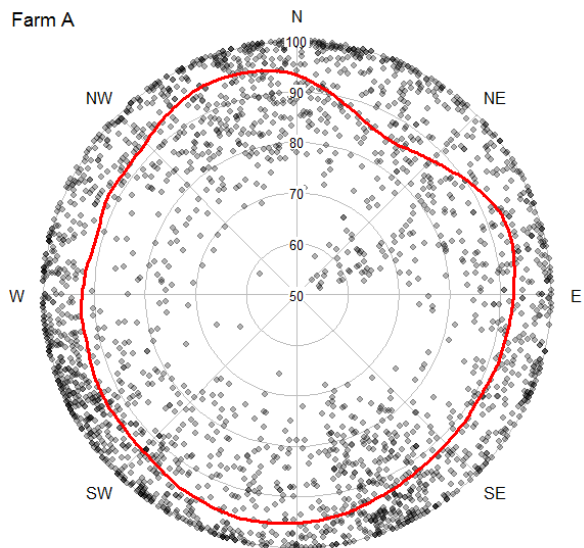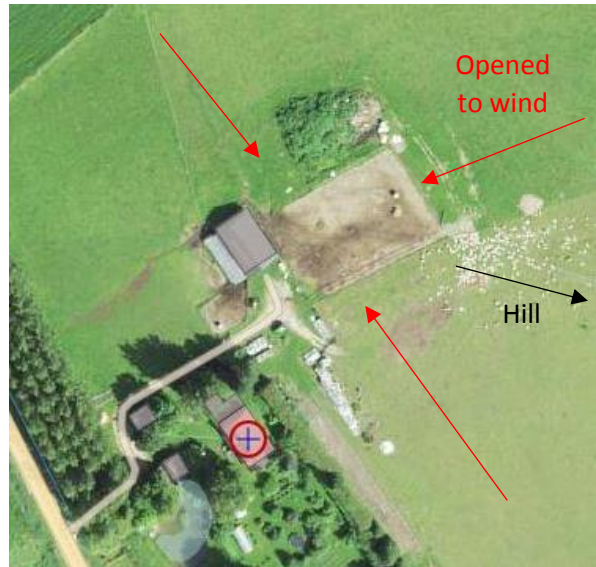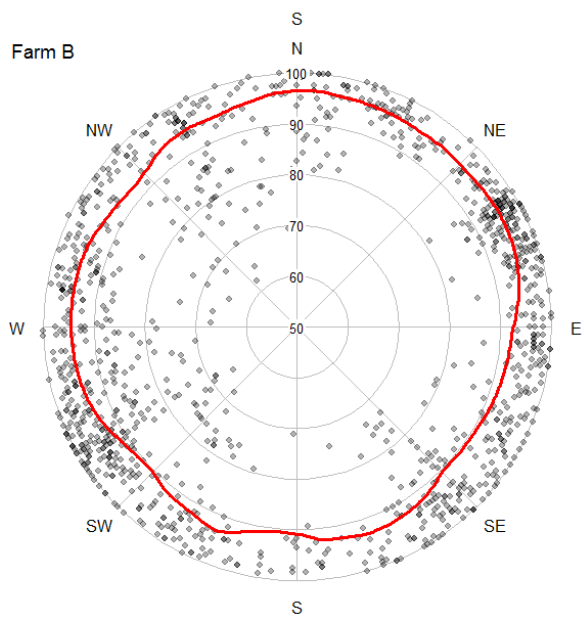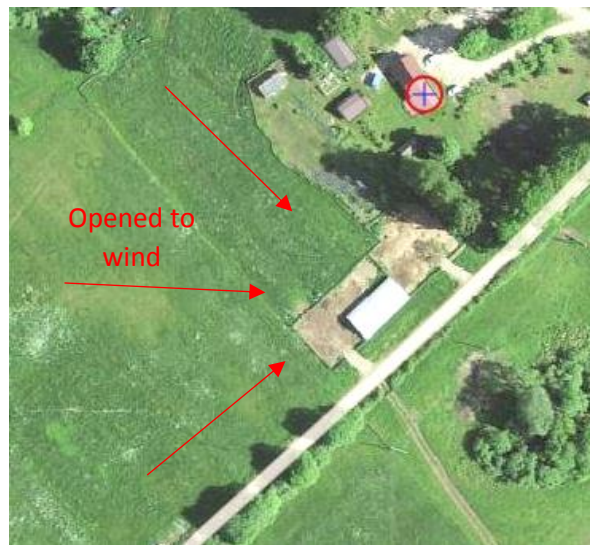

Supplement: Supplementary file 1 [file animals-10-01029-s001.zip › Supplementary/Figure S4.pdf]
